# Anterior gradient protein 2 promotes survival, migration and invasion of papillary thyroid carcinoma cells

**DOI:** 10.1186/1476-4598-13-160

**Published:** 2014-06-30

**Authors:** Gennaro Di Maro, Paolo Salerno, Kristian Unger, Francesca Maria Orlandella, Mario Monaco, Gennaro Chiappetta, Gerry Thomas, Malgorzata Oczko-Wojciechowska, Mariorosario Masullo, Barbara Jarzab, Massimo Santoro, Giuliana Salvatore

**Affiliations:** 1 Dipartimento di Medicina Molecolare e Biotecnologie Mediche, Università di Napoli “Federico II”, Napoli, Italy; 2 Department of Surgery and Cancer, Hammersmith Hospital, Imperial College London, London, UK; 3 UOC Genomica Funzionale, Dipartimento Ricerca, Istituto Nazionale Tumori Fondazione G. Pascale- IRCCS, Napoli, Italia; 4 MSC Memorial Cancer Center and Institute of Oncology, Gliwice, Poland; 5 Dipartimento di Scienze Motorie e del Benessere, Universita’ “Parthenope”, Via Medina 40, Naples 80133, Italy

**Keywords:** Thyroid cancer, AGR2, Endoplasmic reticulum stress, Survival, Migration and invasion

## Abstract

**Background:**

Through a transcriptome microarray analysis, we have isolated Anterior gradient protein 2 (AGR2) as a gene up-regulated in papillary thyroid carcinoma (PTC). AGR2 is a disulfide isomerase over-expressed in several human carcinomas and recently linked to endoplasmic reticulum (ER) stress. Here, we analyzed the expression of AGR2 in PTC and its functional role.

**Methods:**

Expression of AGR2 was studied by immunohistochemistry and real time PCR in normal thyroids and in PTC samples. The function of AGR2 was studied by knockdown in PTC cells and by ectopic expression in non-transformed thyroid cells. The role of AGR2 in the ER stress was analyzed upon treatment of cells, expressing or not AGR2, with Bortezomib and analyzing by Western blot the expression levels of GADD153.

**Results:**

PTC over-expressed AGR2 at mRNA and protein levels. Knockdown of AGR2 in PTC cells induced apoptosis and decreased migration and invasion. Ectopic expression of AGR2 in non-transformed human thyroid cells increased migration and invasion and protected cells from ER stress induced by Bortezomib.

**Conclusions:**

AGR2 is a novel marker of PTC and plays a role in thyroid cancer cell survival, migration, invasion and protection from ER stress.

## Background

Thyroid carcinomas arising from thyroid follicular cells include papillary thyroid carcinoma (PTC), follicular thyroid carcinoma as well as the less common anaplastic thyroid carcinoma [1-3]. PTC is a well-differentiated, slow growing and treatable tumor. The tumor is usually removed by surgery and treated by iodine 131-radiation therapy, however a few PTC carriers develop recurrences and distant metastases [4,5].

To look for genes potentially involved in the neoplastic transformation of the thyroid gland, we conducted a transcriptome microarray screening [6]. Among the genes highly up-regulated in PTC, we focused on Anterior gradient protein 2 (AGR2). AGR2, also known as hAG-2 or Gob-4, is the human orthologue of the *Xenopus laevis* protein XAG-2. In the frog embryo XAG-2 induces cement gland differentiation [7,8]. Several studies have shown a significant function for AGR2 in biological pathways including cell migration and transformation [9,10]. AGR2 protein is up-regulated in several human carcinomas, including breast, pancreatic, ovarian, lung and prostate ones, and is associated with a metastatic phenotype and poor prognosis [11-16].

AGR2 was found up-regulated in several published PTC microarrays [17-21]. Delys and colleagues produced a list of genes modulated in PTC, by comparing their data sets with two independent PTC microarray data sets, and AGR2 scored as one of the genes commonly up-regulated in PTC [19].

Over-expression or suppression of AGR2, in different cancer model systems, affects cell proliferation, invasion, survival and metastasis [9,10].

Recently, AGR2 has been shown to have structural characteristics of the protein disulfide isomerase (PDI) family, including a carboxyl-terminal endoplasmic reticulum (ER) retention signal (KTEL) and a single thioredoxin-like domain with a CXXS motif [22]. PDI proteins catalyze formation, reduction, and isomerization of disulfide bonds, thereby facilitating the maturation of proteins in the ER and ensure correct folding and multimerization of proteins [23,24].

During tumorigenesis, the high proliferation rate of cancer cells requires increased ER protein folding, assembly, and transport, a condition that can induce ER stress [25,26]. Importantly, AGR2 knock out mice showed elevated ER stress [27]. AGR2 expression is induced by ER stress, and siRNA-mediated knockdown of AGR2 increased ER stress response [27,28]. It has been shown that AGR2 exists in monomer/dimer equilibrium and that intermolecular salt bridges involving glutamic acid 60 or cysteine 81 (in the thioredoxin domain of AGR2) stabilize the dimer [29-31]. Importantly, it was demonstrated that dimerization of AGR2 is crucial in mediating the ER stress signaling pathway [29]. AGR2 localizes in the ER of normal intestinal epithelial cells and is essential for *in vivo* production of mucus [32,33]. Indeed, AGR2 mediates processing of the intestinal MUC2 through formation of mixed disulfide bonds [33].

In this work, we analyzed the expression of AGR2 in PTC and its functional role.

## Results

### AGR2 is up-regulated in human PTC samples

**Figure 1:**
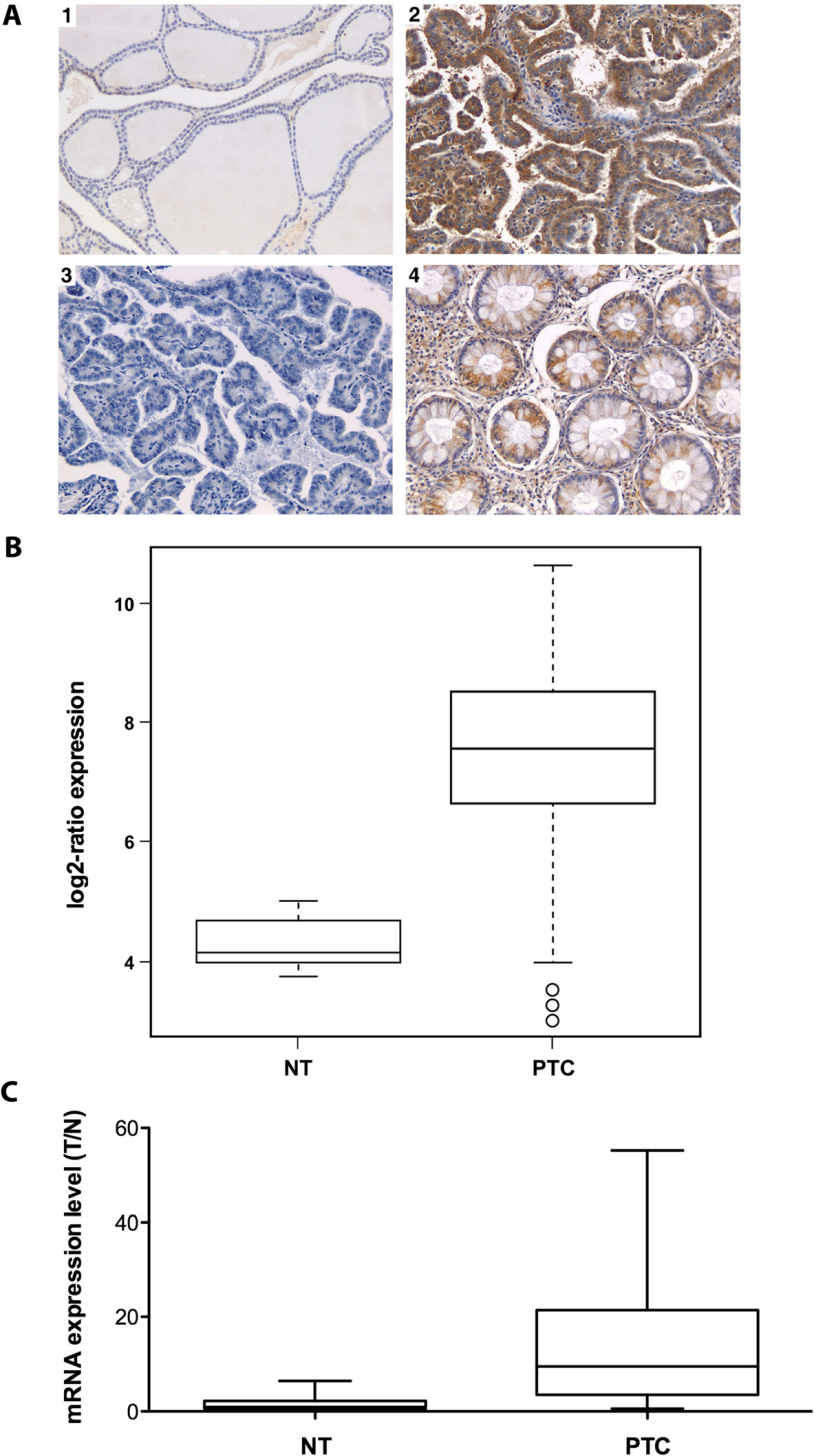
**AGR2 expression in human thyroid tissue samples. A)** Representative images (at 200x magnification) of normal thyroid (NT) (inset 1), papillary thyroid carcinoma (PTC) (inset 2), normal colon sample (inset 4) (used as positive control) stained with a mouse monoclonal anti-AGR2 antibody. PTC shows a strong immunoreactivity for AGR2, whereas NT is negative. Inset 3, shows a PTC sample incubated only with secondary antibody as negative control. **B)** Expression of AGR2 in DNA array of 15 normal thyroid (NT) samples and 43 PTC. The box plot of AGR2 shows a significant up-regulation of this gene in tumor tissues. **C)** Quantitative RT-PCR showing increased levels of AGR2 in PTC samples (n = 10) in comparison to normal thyroid controls (n = 10). The level of AGR2 expression in each sample was measured by comparing its fluorescence threshold with the average fluorescence threshold of the NT samples.

We measured AGR2 expression by immunohistochemistry with an anti-AGR2 monoclonal antibody in 64 samples including 25 normal thyroid (NT) samples and 39 PTC samples. Representative immunohistochemical staining is shown in Figure 1A, and the entire dataset is reported in Table 1. AGR2 was expressed at low levels in normal thyroid glands (Figure 1A, inset 1). In contrast, several PTC samples (PTC classical variant: 23 out of 28 samples; PTC follicular variant: 5 out of 11 samples) were strongly positive for AGR2 expression, and positivity was confined to tumor cells (Table 1 and Figure 1A, inset 2). No staining was observed in the absence of the primary antibody (Figure 1A, inset 3). Normal colon mucosa was used as positive control (Figure 1A, inset 4).

**Table 1:** AGR2 expression in thyroid samples (n = 64)

Tissue*	Number of samples**	AGR2 (score***)			
		**Negative**	**Positive**		
		**0**	**1+**	**2+**	**3+**
NT	25	4	21		
PTC	28 (PTC CV)	1	2	2	23
	11 (PTC FV)	2	1	3	5

*NT = normal thyroid; PTC = papillary thyroid carcinoma.

**PTC CV = papillary thyroid carcinoma, classical variant; PTC FV = papillary thyroid carcinoma, follicular variant.

***0 = < 10% of positive cells; 1 = 10-50% of positive cells; 2 = 51-75% of positive cells; 3 = 76-100% of positive cells.

To determine whether up-regulation occurred also at RNA level, we verified AGR2 expression levels in a DNA array dataset of 15 normal human thyroid (NT) and 43 PTC samples. As shown in Figure 1B, PTC samples showed significantly increased AGR2 levels compared to normal thyroid tissues (*P* < 0.0001). These findings were further confirmed by quantitative RT-PCR analysis of an independent set of PTC samples (Figure 1C).

### Knockdown of AGR2 induces apoptosis of TPC-1 cells

**Figure 2:**
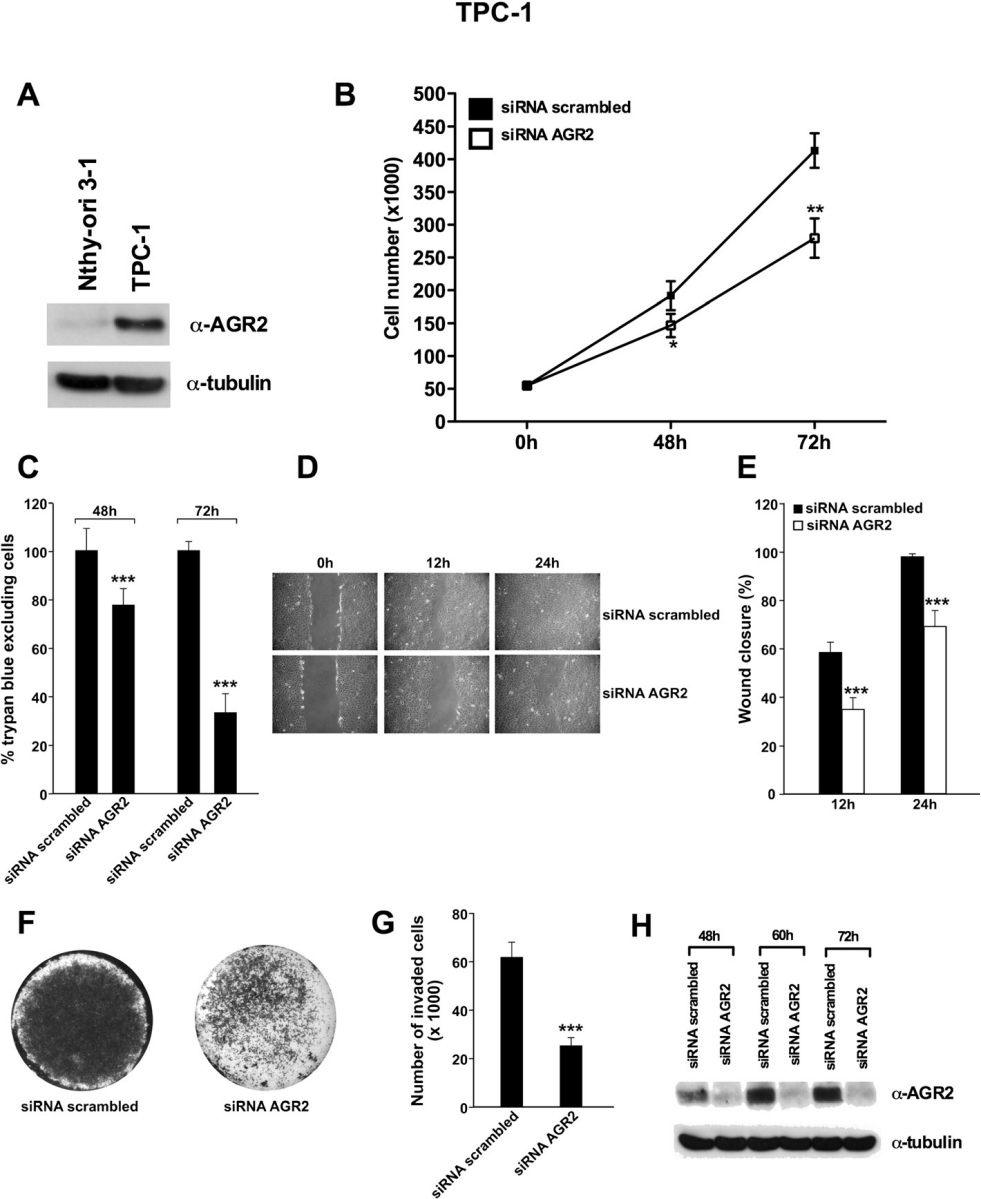
**Effects of AGR2 knockdown on TPC-1 cell growth, migration and invasion. A)** Expression levels of AGR2 in Nthy-ori 3–1 and TPC-1 cells. Cells were lysed and analyzed by Western blotting with the indicated antibodies. **B)** TPC-1 cells were transfected with AGR2 siRNA or with scrambled siRNA and counted at different time points. Values represent the average of triplicate experiments ± standard deviations. **C)** TPC-1 cells were transfected with AGR2 siRNA or with scrambled siRNA; after 48 and 72 hours cells were collected by trypsinization, stained for 10 minutes with trypan-blue and counted in triplicate. The percentage of trypan blue excluding cells compared to cells transfected with scrambled siRNA is reported ± standard deviations. **D)** TPC-1 cells were transfected with AGR2 siRNA or with scrambled siRNA; after 48 hours a wound was introduced and cell migration into the wound was monitored at 12 and 24 hours. **E)** Wound closure was measured by calculating pixel densities in the wound area and expressed as percentage of wound closure of triplicate areas ± standard deviations. **F)** TPC-1 (1 × 10^5^) cells were transfected with AGR2 siRNA or with scrambled siRNA; 48 hours after transfection, cells were seeded in the upper chamber of transwells and incubated for 24 hours; the upper surface of the filter was wiped clean and cells on the lower surface were stained, photographed and counted. This figure is representative of three independent experiments. **G)** Invasive ability is expressed as number of invaded cells. Values represent the average of triplicate experiments ± standard deviations. **H)** TPC-1 cells were transfected with AGR2 siRNA or with scrambled siRNA. Cells were harvested at different time points and protein lysates were subjected to immunoblotting with the indicated antibodies. Asterisks indicate *P* < 0.05 (*), *P* <0.01 (**) and *P* < 0.001 (***).

We evaluated the expression level of AGR2 in the papillary thyroid cancer cell line TPC-1 in comparison to non-transformed thyroid cells Nthy-ori 3–1. As shown in Figure 2A, AGR2 is over-expressed in TPC-1 cells. We evaluated the effects of AGR2 ablation in TPC-1 cells by RNA interference. TPC-1 cells were transfected with AGR2 siRNA or with scrambled siRNA, counted and lysed at different time points. Forty-eight hours after transfection TPC-1 cells transfected with scrambled siRNA numbered 192 × 10^3^, whereas those transfected with AGR2 siRNA numbered 146 × 10^3^ (*P =* 0.0492). Seventy–two hours after transfection, TPC-1 cells transfected with scrambled siRNA numbered 413 × 10^3^, whereas those transfected with AGR2 siRNA numbered 279 × 10^3^ (*P =* 0.0045) (Figure 2B). Accordingly, the fraction of trypan blue excluding (viable) cells of TPC-1 transfected with AGR2 siRNA, was reduced with respect to scrambled control, at 48 and 72 hours post transfection (*P* < 0.0001) (Figure 2C).

### Knockdown of AGR2 reduces migration and invasion of TPC-1 cells

We evaluated migration (by a wound-closure assay) and invasion (by a Matrigel invasion assay) ability of AGR2 siRNA-transfected cells. A scraped wound was introduced on a confluent monolayer of TPC-1 cells transfected with AGR2 siRNA or scrambled siRNA. Figure 2D and E show that TPC-1 transfected with the scrambled control efficiently migrated into the wound; in contrast, cells transfected with AGR2 siRNA had a reduced migrating ability both at 12 and 24 hours (*P* < 0.0001). Moreover, cells transfected with AGR2 siRNA showed a reduced ability to invade Matrigel compared to control cells (*P* < 0.0001) (Figure 2F and G). The efficiency of AGR2 knockdown at the different time points examined is shown in Figure 2H.

### Ectopic AGR2 promotes migration and invasion of Nthy-ori 3–1 cells

**Figure 3:**
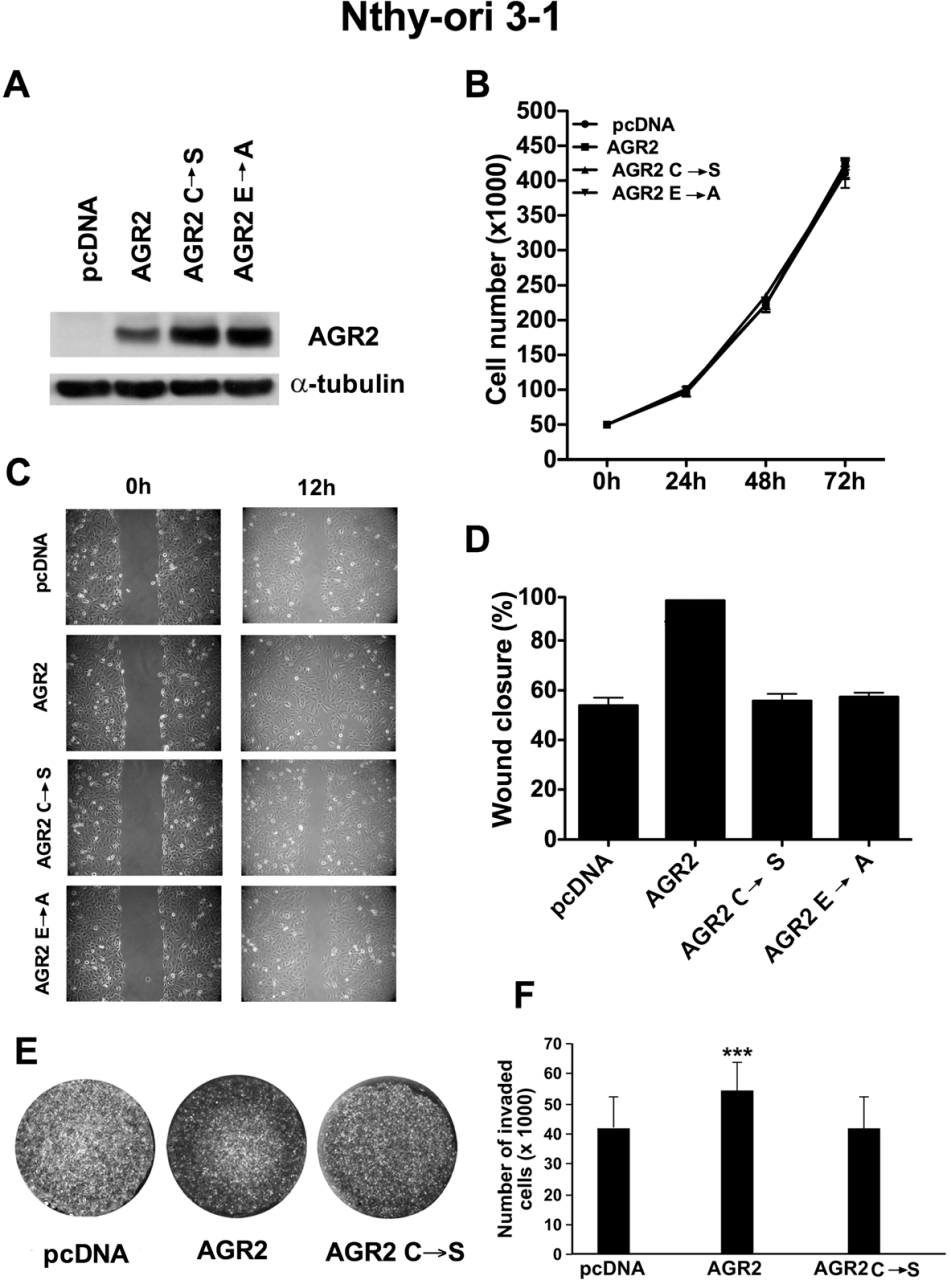
**Effects of AGR2 ectopic expression on Nthy-ori 3–1 cell growth, migration and invasion. A)** Expression levels of AGR2, AGR2 (C → S) and AGR2 (E → A) in transfected Nthy-ori 3–1 cells: after G418 selection, cells were lysed and analyzed by Western blotting with the indicated antibodies. **B)** Nthy-ori 3–1 cells transfected with AGR2, AGR2 (C → S), AGR2 (E → A) or empty vector (pcDNA) were plated and counted at different time points. Values represent the average of triplicate experiments ± standard deviations. **C)** A wound was introduced on confluent monolayer of Nthy-ori 3–1 cells transfected with AGR2, AGR2 (C → S), AGR2 (E → A) and vector control (pcDNA) and wound closure was monitored at 12 hours time points. **D)** Wound closure was measured by calculating pixel densities in the wound area and expressed as percentage of wound closure of triplicate areas ± standard deviations. **E)** Nthy-ori 3–1 cells transfected with AGR2, AGR2 (C → S) or the empty vector (pcDNA) were seeded in the upper chamber of transwells and incubated for 24 hours; the upper surface of the filter was wiped clean and cells on the lower surface were stained and counted. **F)** Invasive ability was expressed as number of invaded cells. Values represent the average of triplicate experiments ± standard deviations. Asterisks indicate *P* < 0.001 (***).

We transfected the non-transformed thyroid cells, Nthy-ori 3–1, with the empty vector (pcDNA), AGR2 wild type, AGR2-carrying the cysteine 81 to serine mutation AGR2 (C → S), or AGR2 carrying the glutamic acid 60 to alanine mutation AGR2 (E → A) (Figure 3A). Wild type AGR2, after treatment with disuccinimidyl suberate (DSS), is dimeric (Additional file 1: Figure S1). The C81S mutation targets the thioredoxin domain of AGR2 and impairs its catalytic activity and dimer formation, while the E60A mutation impairs AGR2 dimer formation [29-31]. Nthy-ori 3–1 AGR2, AGR2 (C → S), AGR2 (E → A) and pcDNA cells showed comparable growth rates (Figure 3B). A scraped wound was introduced on the confluent monolayer and cell migration into the wound was monitored after 12 hours. As shown in Figure 3C and D, migration rate was larger in AGR2-transfected cells compared to control vector (pcDNA), AGR2 (C → S) and AGR2 (E → A) cells. Then we seeded AGR2, AGR2 (C → S) and vector control (pcDNA) cells into the top chamber of transwells and evaluated their ability to invade Matrigel. AGR2 over-expression increased the invasive ability of Nthy-ori 3–1 cells in comparison to vector control (*P* < 0.0001) and AGR2 (C → S) cells (*P* < 0.0001) (Figure 3E and F).

### Ectopic AGR2 promotes cell migration and invasion of TPC-1 cells

To confirm these findings, we over-expressed AGR2 in TPC-1 cells. Cells were transfected with AGR2, AGR2 (C → S), AGR2 (E → A) or with the empty vector (pcDNA). Mass populations were selected in G418 (Additional file 1: Figure S2A). Growth rates of TPC-1 AGR2, AGR2 (C → S), AGR2 (E → A) and control (pcDNA) cell lines were similar (Additional file 1: Figure S2B). We studied cell migration using the wound closure assay. As shown in Additional file 1: Figure S2C, migration rate was larger in TPC-1 AGR2 cells compared to AGR2 (C → S), AGR2 (E → A) cells and to vector control (pcDNA) cells. We next seeded TPC-1 AGR2, AGR2 (C → S), and pcDNA control cells into the top chamber of transwells and evaluated their ability to invade Matrigel. AGR2 over-expression increased TPC-1 invasiveness in comparison to AGR2 (C → S) cells and control vector (Additional file 1: Figure S2D).

### Nthy-ori 3–1 AGR2 cells have an increased disulfide isomerase activity

**Figure 4:**
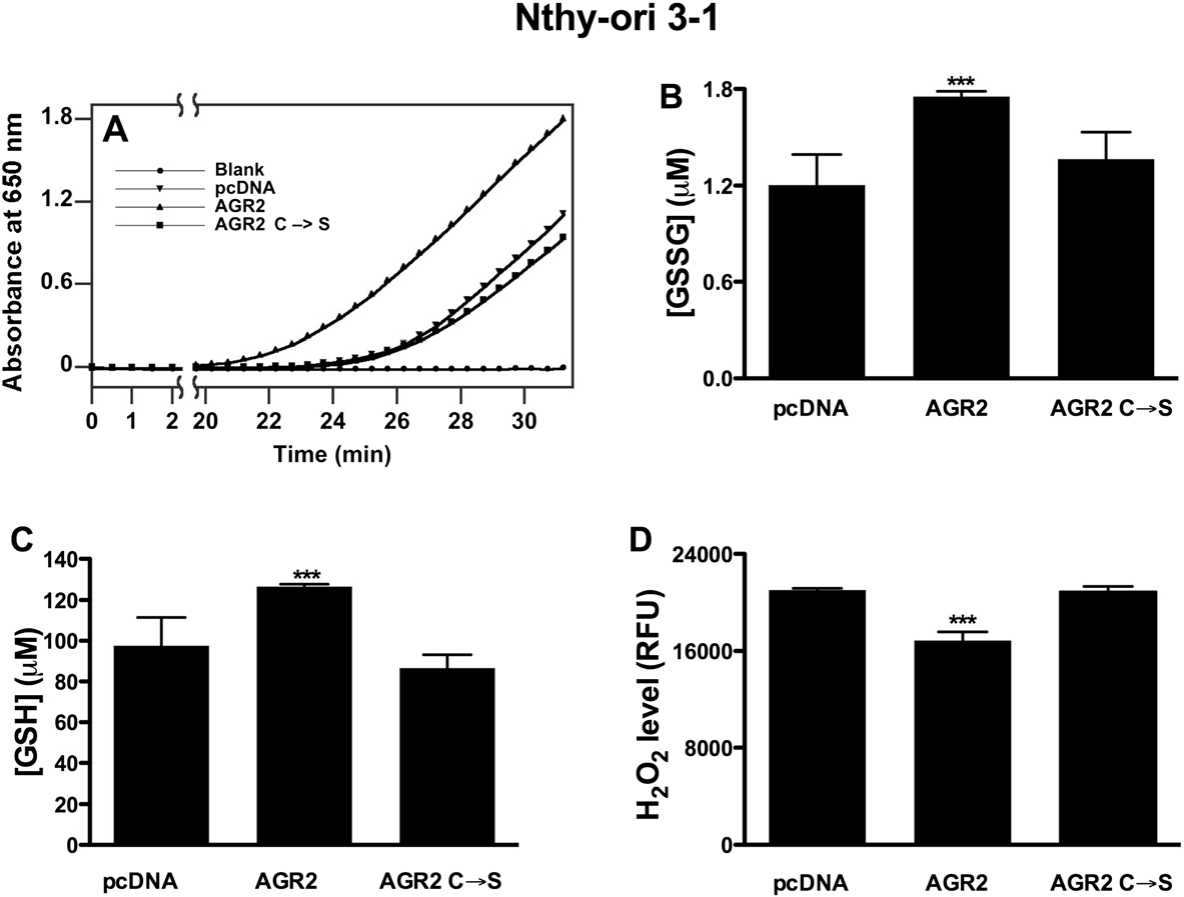
**Disulfide isomerase activity of AGR2 and intracellular redox state. A)** The disulfide isomerase activity of AGR2 was assayed with the insulin precipitation method. The absorbance at 650 nm was plotted against time (minutes). Only dithiothreitol was used as blank. **B-C)** Levels of GSSG and GSH in Nthy-ori 3–1 AGR2, AGR2 (C → S) and vector control cells were determined using GSH and GSSG assay kit. Values represent the average of duplicate experiments ± standard deviations. **D)** Nthy-ori 3–1 AGR2, AGR2 (C → S) and vector control cells were incubated H_2_DCFDA (100 μM) and levels of H_2_O_2_ were measured as described in Methods. Asterisks indicate *P* < 0.001 (***).

AGR2 has been identified as a protein disulfide isomerase, playing a role in the endoplasmic reticulum (ER) stress response [22-33]. Protein disulfide isomerases catalyze the formation (oxidation), breakage (reduction) and rearrangement (isomerization) of disulfide bonds between cysteine residues within proteins during their folding process. To determine whether AGR2 had a disulfide isomerase activity in thyroid cells we performed an insulin-reduction assay. In this assay, insulin reduction catalyzed by protein disulfide isomerases in the presence of dithiothreitol (DTT) results in the aggregation of its β chains that can be spectrophotometrically revealed at 650 nm [34]. As shown in Figure 4A, Nthy-ori 3–1 AGR2 cells showed an increased rate of insulin reduction compared to AGR2 (C → S) or vector control cells.

To confirm these findings, we measured the levels of oxidized (GSSG) and of reduced (GSH) glutathione in Nthy-ori 3–1 AGR2, AGR2 (C → S) and vector control cells. As shown in Figure 4B and C, levels of GSSG and GSH were increased in Nthy-ori 3–1 AGR2 cells compared to AGR2 (C → S) and to vector control cells. Finally, as shown in Figure 4D Nthy-ori 3–1 AGR2 cells displayed a decreased level of H_2_O_2_ compared to vector control and to AGR2 (C → S) cells (*P <* 0.001).

### AGR2 protects non-transformed thyroid Nthy-ori 3–1 cells from endoplasmic reticulum stress

**Figure 5:**
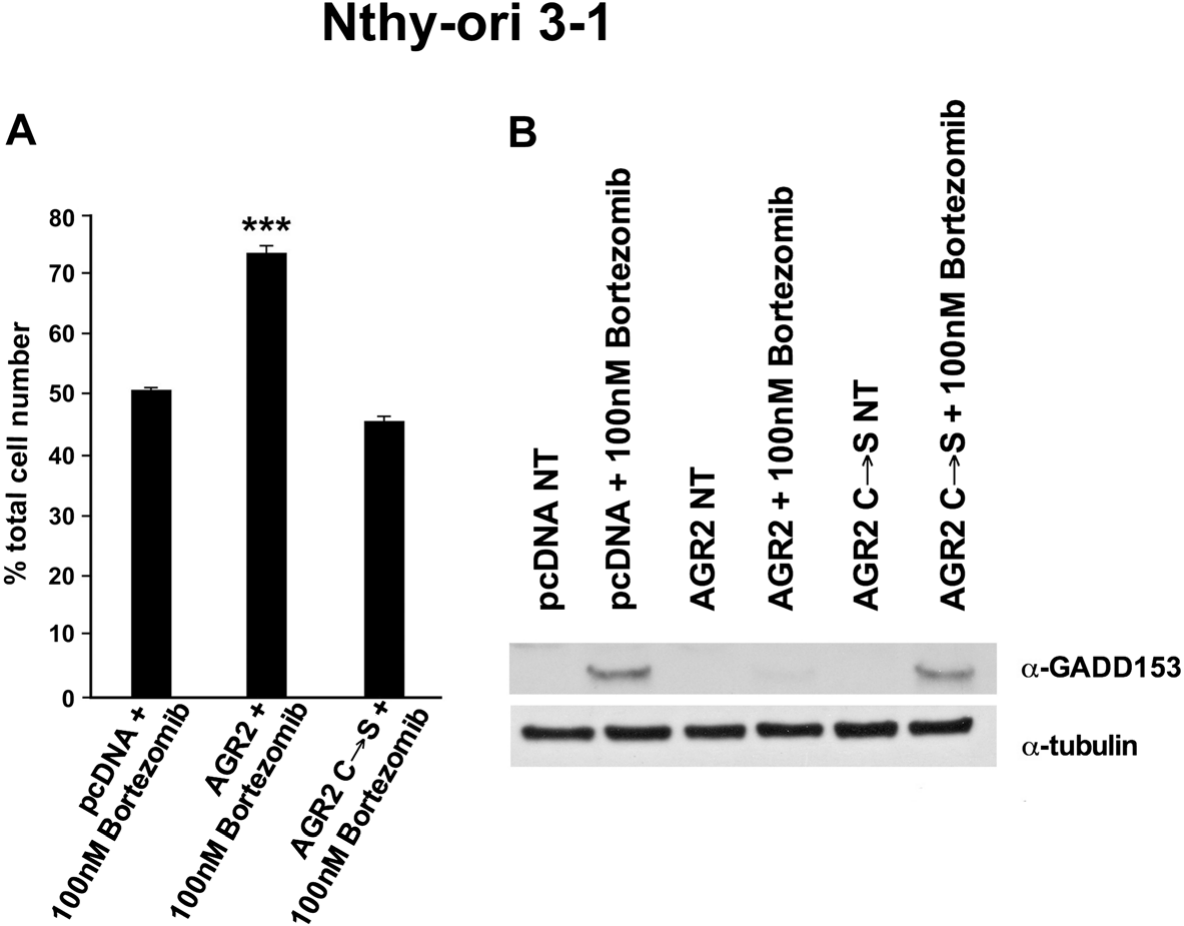
**Effects of Bortezomib in Nthy-ori 3–1 cells ectopically expressing AGR2. A)** Nthy-ori 3–1 cells transfected with AGR2, AGR2 (C → S) or with the empty vector (pcDNA) were treated for 24 hours with Bortezomib 100 nM. After treatment, cells were collected by trypsinization and counted in triplicate. The percentage of total cell number respect to untreated cells is reported ± standard deviations. **B)** Nthy-ori 3–1 cells transfected with AGR2, AGR2 (C → S) or with the empty vector (pcDNA) were treated for 24 hours with Bortezomib 100 nM. After treatment, cells were lysed, and protein lysates were subjected to immunoblotting with the indicated antibodies. Asterisks indicate *P* < 0.001 (***).

It was shown that AGR2 has a role in the endoplasmic reticulum (ER) stress response [27-30]. Thus, we studied the effects of ectopic AGR2 expression in the ER stress pathway upon treatment with Bortezomib. Bortezomib is a potent and selective inhibitor of the proteasome that causes an accumulation of unfolded proteins in the ER [35-37]. Nthy-ori 3–1 cells transfected with AGR2, AGR2 (C → S), or vector (pcDNA) were treated for 24 hours with 100 nM Bortezomib. After treatment, cells were counted. As shown in Figure 5A, AGR2 protected Nthy-ori 3–1 cells from reduced viability caused by Bortezomib in comparison to AGR2 (C → S) and to control (pcDNA) cells.

Then, we determined by Western blot the expression level of GADD153 (also named Chop), a well-characterized biomarker of ER stress [37], in Nthy-ori 3–1 AGR2, AGR2 (C → S) or empty vector (pcDNA) cells treated with Bortezomib (100 nM) for 24 hours. As shown in Figure 5B, Nthy-ori 3–1 AGR2 cells presented a blunted increase of GADD153 upon Bortezomib treatment compared to pcDNA and to AGR2 (C → S) cells. These data suggest that ectopic AGR2 expression protects non-transformed thyroid cells from ER stress induced by Bortezomib.

## Discussion

AGR2 is a protein disulfide isomerase that is over-expressed in several human carcinomas and stimulates cancer cell proliferation, survival, invasion, and metastasis [9,10]. In the present study, we demonstrated that AGR2 is over-expressed in PTC. Our data are in agreement with thyroid carcinoma microarray screenings that isolated AGR2 as a gene up-regulated in PTC [17-21].

We investigated the functional role of AGR2 using gain and loss-of-function approaches. Knockdown of AGR2 in PTC cells decreased cell survival, migration and invasion. On the other hand, forced AGR2 expression in non-transformed thyroid Nthy-ori 3–1 cells, resulted in increased cell migration and invasion and protection from ER stress.

Proteins that fail to fold properly are retained in the ER and their accumulation may constitute a form of stress to the cell. Several signaling pathways, collectively known as unfolded protein response (UPR), have evolved to detect the accumulation of misfolded proteins in the ER and activate a cellular response to maintain homeostasis [25,26]. As a member of the protein disulfide isomerase family, AGR2 aids protein folding and assembly by catalyzing the formation, reduction, and isomerization of disulfide bonds, thereby stabilizing intermediate conformations during protein maturation in the ER.

Accordingly, here we demonstrated *in vitro* that AGR2 acts as a disulfide isomerase in Nthy-ori 3–1 cells. By evaluating the aggregation of insulin as a result of reduction of disulfide bonds catalyzed by protein disulfide isomerase, we demonstrated that AGR2 over-expressing cells have an increased disulfide isomerase activity compared to control cells, an increased levels of GSSG and GSH and a decreased level of H_2_O_2_.

Further, we showed that wild type AGR2 is dimeric in transfected Nthy-ori 3–1 cells treated with DSS. Interestingly, it was recently demonstrated that dimerization of AGR2 attenuates ER stress-induced cell death through the association with BiP/GRP78 [29-31].

Increased expression of the UPR components, including GADD153 has been detected in breast, lung, gastric, and esophageal carcinomas [38-40]. In this context, our hypothesis is that AGR2 is up-regulated by PTC cells to cope with the ER stress. Thus, as shown for other ER resident proteins [25,26], increased AGR2 expression in PTC could enhance ER folding capacity and allow cancer cells to cope with increased protein production and secretion [41,42].

## Conclusions

Here, we demonstrated that AGR2 plays a key role in thyroid cancer cell survival, migration, invasion and protection from ER stress. AGR2 may be one of the pro-survival factors used by cancer cells to overcome stress due to excess protein production and to assist protein folding, degradation or both. These findings suggest that AGR2 could be exploited as a molecular marker of PTC.

## Methods

### Tissue samples

Tumor (n = 39) and normal (n = 25) thyroid tissue samples for immunohistochemical analysis were retrieved from the Pathology Department of the Istituto Pascale, Naples, Italy. In addition, RNA from frozen tissues of 10 PTC cases and 10 normal matched thyroid controls was obtained from the Chernobyl Tissue Bank (http://www.chernobyltissuebank.com) [43]. A set of 43 PTC and 15 normal thyroid tissues, also obtained from Chernobyl Tissue Bank, was used in the DNA array study (HG-U133 Plus 2.0, Handkiewicz Junak et al., manuscript submitted for publication) carried out in MSC Memorial Cancer Center and Institute of Oncology, Gliwice, Poland. In all cases, the respective Institutional review boards approved the study. The samples were classified according to the diagnostic criteria required for the identification of PTC [44].

### Immunohistochemical staining

Sections of paraffin-embedded samples were stained with hematoxylin and eosin for histological examination [44]. For immunohistochemistry, 2 μm paraffin sections were deparaffinized and placed in a solution of 0.3% hydrogen peroxide in absolute methanol for 30 minutes and then washed in phosphate buffered saline (PBS) before immunoperoxidase staining. The slides were then incubated overnight at 4°C in a humidified chamber with mouse monoclonal antibody against AGR2 (PO1, Abnova, Taipei City, Taiwan) diluted 1:100 in PBS. The slides were subsequently incubated with biotinylated goat anti-mouse IgG for 20 minutes (Vectostain ABC kits, Vector Laboratories) and then with premixed reagent ABC (Vector) for 20 minutes. The immunostaining was performed by incubating the slides in diaminobenzidine (DAB, DAKO) solution containing 0.06 mM DAB and 2 mM hydrogen peroxide in 0.05% PBS, pH 7.6, for 5 minutes, and after chromogen development, the slides were washed, dehydrated with alcohol and xylene and mounted with coverslips using a permanent mounting medium (Permount). Micrographs were taken on Kodak Ektachrome film with a Zeiss system. Negative controls were performed in each case by incubating tissue slides with secondary antibody only. The expression of AGR2 was categorized as positive (staining of ≥10% of the tumor cells) or negative (staining of < 10% of the tumor cells). The staining intensity was further graded in: score 1 (10-50% of positive cells), score 2 (51-75% of positive cells) and score 3 (76-100% of positive cells). Score values were independently assigned by two blinded investigators (G.C. and M.M.) and a consensus was reached on all scores used for computation.

### Cell cultures

Human papillary thyroid cancer cell line TPC-1 was obtained from M. Nagao (Carcinogenesis Division, National Cancer Center Research Institute, Tokyo, Japan) and identified based on the presence of the RET/PTC1 rearrangement. TPC-1 cell line was grown Dulbecco’s modified Eagle’s medium (DMEM) (Invitrogen) supplemented with 10% fetal bovine serum (FBS), L-glutamine and penicillin/streptomycin (Invitrogen). Nthy-ori 3–1 cells are non-transformed human thyrocytes immortalized by the Large T of SV40 and were obtained from the European Tissue Culture collection (Sigma Aldrich, St. Louis, MO, USA). Nthy-ori 3–1 cell line was grown in Roswell Park Memorial Institute (RPMI) 1640 medium supplemented with 10% fetal bovine serum (FBS), L-glutamine and penicillin/streptomycin (Invitrogen, Carlsbad, California, USA).

### Reagents

Bortezomib was obtained from Millenium (Cambridge, MA, USA). Human insulin, disuccinimidyl suberate (DSS) and dithiothreitol (DTT) were from Sigma Aldrich (St. Louis, MO, USA). Glutathione reduced and oxidized assay kit was obtained from Bioassay Systems (Hayward, CA, USA).

### Cell viability

For cell viability determination, cells were collected by trypsinization stained for 10 minutes with 0.4% trypan-blue (Sigma Aldrich) according to manufacturer’s instructions, and counted in triplicate.

### Chemoinvasion

Cell invasion was examined using a reconstituted extracellular matrix (Matrigel, BD Biosciences, San Jose, CA). The cell suspension (1 × 10^5^ cells per well) was added to the upper chamber of transwell cell culture chambers on a prehydrated polycarbonate membrane filter of 8 μm pore size (Costar, Cambridge, MA) coated with 35 μg of Matrigel (BD Biosciences). The lower chamber was filled with 10% medium. After incubation at 37°C, non-migrating cells on the upper side of the filter were wiped-off. Invading cells were mounted on glass slides using mounting medium and stained with Hoechst (Sigma Aldrich). Cell migration was quantified by counting the number of stained nuclei in three individual fields in each transwell membrane, by fluorescence microscopy, in duplicate.

### Wound closure assay

A wound was introduced on the confluent monolayer cells using a micropipette tip. Photographs were taken at 40 X magnification using phase-contrast microscopy immediately after wound incision and at selected time-points. Wound closure was measured by calculating pixel densities in the wound area by Cell^a^ software (Olympus Biosystem Gmb, Hamburg, Germany) and expressed as percentage of wound closure of triplicate areas ± SD.

### RNA silencing

A pool of 4 small inhibitor duplex RNAs (ON-*TARGETplus* siRNA SMARTpool) targeting human AGR2 (#L-003626-00), and a non-targeting pool (ON-*TARGETplus* Non-Targeting Pool) control (#D 001810-10-20) were purchased from Dharmacon RNAi Technologies (Dharmacon Inc., Chicago, IL, USA). Cells were grown under standard conditions. TPC-1 cells were plated in 60-mm dishes in complete medium without antibiotics and electroporated using MicroPorator (MP-100, Digital Bio, Euroclone, Milan, Italy) according to the manufacturer’s instructions. Cells were harvested at different time points after transfection, counted and analyzed for protein expression.

### Protein studies

Immunoblotting was carried out according to standard procedures. Anti-AGR2 (PO1) monoclonal antibody was from Abnova (Taipei City, Taiwan); anti-GADD153 (B-3, sc-7351) and anti-α-tubulin monoclonal antibody was from Sigma-Aldrich. Secondary anti-mouse and anti-rabbit antibodies coupled to horseradish peroxidase were from Santa Cruz Biotechnology.

### *Generation of stable* AGR2 *transfectants*

cDNA containing the complete coding sequence of human AGR2 was produced by reverse transcription of RNA from HT29 colon adenocarcinoma cells (American Type Culture Collection, Rockville, MD, USA) using the following primers:

AGR2 Cl For: 5′-ATCCTCGAGCGATCATGGAGAAAATTCCA-3′;

AGR2 Cl Rev: 5′-ATCGGATCCCAATTCAGTCTTCAGCAA-3′ (annealing temperature: 60°C).

AGR2 cDNA was cloned into pcDNA3.1 (named pcDNA) (Invitrogen) by using BamHI and XhoI restriction enzymes and verified by sequencing.

Point mutations in AGR2 that changed the cysteine at position 81 to serine (C → S) and glutamic acid at position 60 to alanine (E → A) were introduced in the pcDNA-AGR2 plasmid by the Stratagene QuikChange site-directed mutagenesis kit (Agilent Technologies, Inc. Life Sciences and Chemical Analysis Group, Santa Clara, CA, USA).

The following oligonucleotides were used:

AGR2 C81S For: 5′- CATCACTTGGATGAGTCCCCACACAGTCAAGC-3′;

AGR2 C81S Rev: 5′-GCTTGACTGTGTGGGGACTCATCCAAGTGATG-3′ (annealing temperature: 55°C).

AGR2 E60A For: 5′- CTGGACTCAGACATATGAAGCAGCTCTATATAAATCCAAGAC-3′;

AGR2 E60A Rev: 5′-GTCTTGGATTTATATAGAGCTGCTTCTTCATATGTGTCCAG-3′ (annealing temperature: 55°C).

The Nthy-ori 3–1 and the TPC-1 cells were transfected with pcDNA-AGR2, AGR2 (C → S), AGR2 (E → A) or the empty vector (pcDNA) using the Lipofectamine Reagent (Invitrogen) according to the instructions of the manufacturer. Two days later, G418 (Invitrogen) was added at concentration of 0.3 mg/ml (Nthy-ori 3–1) or of 1.2 mg/ml (TPC-1). Several clones and mass populations of transfectants were isolated, expanded and screened for AGR2 expression by Western blotting.

### Chemical crosslinking

Nthy-ori 3–1 AGR2 transfected cells were treated with 1 mM disuccinimidyl suberate (DSS). Cells were harvested and washed 3 times with ice-cold PBS. The cross-linked cells were incubated for 60 minutes with mild shaking at room temperature, and then quenched by quenching buffer (1 M Tris, pH 7.5) to a final concentration of 10 mM for 15 min at room temperature to stop the chemical reaction. The chemically cross-linked cells were subjected to Western blotting analysis.

### Evaluation of GSSG, GSH and H_2_O_2_ levels

To measure the oxidized glutathione (GSSG) and reduced glutathione (GSH), the EnzyChrom™ GSH and GSSG Assay Kit (EGTT-100) (Bioassay Systems, Hayward, CA, USA) was used according to manufacturer’s instructions. Intracellular hydrogen peroxide levels were determined using the cell-permeable probe 2′,7′-dichlorodihydrofluorescein diacetate (H_2_DCFDA) that upon cleavage of the acetate groups by intracellular esterases and oxidation is converted to 2′,7′-dichlorofluorescein (DCF) (Invitrogen, Carlsbad, CA). DCF is a highly fluorescent compound that can be detected by fluorescence spectroscopy using excitation and emission wavelength of 485 nm and 538 nm, respectively. Cells were incubated with H_2_DCFDA (100 μM) in complete media for 1 hour at 37°C. After that, cells were washed twice in PBS and fluorescence intensity was quantified by microplate reader (Perkin-Elmer Envision 2103 multilabel reader).

### Insulin reduction assay

The disulfide isomerase activity was measured by the reduction of disulfide bonds in human insulin in the presence of DTT, giving rise to the aggregation of its β chain, a process that can be followed by turbidimetry [32]. The assay mixture was prepared in a cuvette by addition of 60 μl of insulin (10 mg/ml) to cell lysates in a buffer containing 100 mM sodium EDTA pH7 and 100 mM sodium phosphate pH 7 to give a final volume of 500 μl. The reaction started with the addition of 2 mM DTT and was followed kinetically by the increase of absorbance at 650 nm, due to insulin β chain precipitation. The absorbance at 650 nm was plotted.

### RNA extraction and expression studies

Total RNA was isolated with the RNeasy Kit (Qiagen, Crawley, West Sussex, UK). The quality of the RNAs was verified by the 2100 Bioanalyzer (Agilent Technologies, Waldbronn, Germany); only samples with RNA integrity number (RIN) value > 7 were used for further analysis. One μg of RNA from each sample was reverse-transcribed with the QuantiTect® Reverse Transcription (Qiagen).

### Quantitative RT-PCR

Quantitative RT-PCR was applied to study AGR2 expression in PTC samples (n = 10) and normal thyroid control (n = 10). RNA from each sample was reverse-transcribed with the QuantiTect® Reverse Transcription (Qiagen). For quantitative RT-PCR, we used the Human ProbeLibray™ system (Roche). AGR2 and RNA polymerase 2 primers sequences were:

AGR2 For: 5′-CTG GCC AGA GAT ACC ACA GTC-3′;

AGR2 Rev: 5′-AGT TGG TCA CCC CAA CCT C-3′;

RNA pol-For: 5′-GCGATGAGAACAAGATGCAA-3′;

RNA pol-Rev: 5′-CGCAGGAAGACATCATCATC-3′.

PCR reactions were performed in triplicate and fold changes were calculated with the formula: 2^-(sample 1 ΔCt - sample 2 ΔCt)^, where ΔCt is the difference between the amplification fluorescent thresholds of the mRNA of interest and the mRNA of RNA polymerase 2 used as an internal reference. Sample 1 represented each single PTC sample, and sample 2 was the average of all (n = 10) normal thyroid samples.

### Statistical analysis

Data are presented as mean ± standard deviations. Two-tailed unpaired Student’s t test was used for all statistical analysis. Mann–Whitney *U* test was used only for DNA array data analysis. *P* < 0.05 was considered statistically significant. Statistical analyses were carried out using the Graph Pad InStat (version 3.1a, La Jolla, CA, USA).

## Abbreviations

AGR2: Anterior gradient protein 2; DCF: 2′,7′-dichlorofluorescein; DSS: Disuccinimidyl suberate; DTNB: 5,5′-dithiobis (2-nitrobenzoic) acid; DTT: Dithiothreitol; ER: Endoplasmic reticulum; GSH: Reduced glutathione; GSSG: Oxidized glutathione; H_2_DCFDA: 2′,7′-dichlorodihyfluorescein diacetate; NT: Normal thyroid; PDI: Protein disulfide isomerase; PTC: Papillary thyroid carcinoma; UPR: Unfolded protein response.

## Competing interests

None of the authors of this manuscript had any conflict of interest regarding the study.

## Authors’ contributions

GDM, FMO, MRM and PS performed functional experiments. MM and GC performed immunohistochemistry studies; KU, GT, MOW and BJ contributed to the expression studies. GDM, MS and GS designed the experiments. All authors read and approved the final manuscript.

## Supplementary Material

Additional file 1: Figure S1 The chemical crosslinker DSS was added to Nthy-ori 3-1 AGR2 cell suspension at final concentration of 1 mM. Monomeric and dimeric AGR2 were detected by Western blotting with AGR2 antibody. Tubulin levels were used for normalization. **Figure S2.** Effects of AGR2 ectopic expression on TPC-1 cell growth, migration and invasion. A) Expression levels of transfected AGR2, AGR2 (C→S) and AGR2 (E→A) in TPC-1 cells: after G418 selection, cells were lysed and blotted with the indicated antibodies. Levels of exogenous AGR2 are shown. B) TPC-1 cells transfected with AGR2, AGR2 (C→S), AGR2 (E→A) or empty vector (pcDNA) were plated and counted at different time points. Values represent the average of triplicate experiments ± standard deviations. C) A wound was introduced on confluent monolayer of TPC-1 cells transfected with AGR2, AGR2 (C→S), AGR2 (E→A) or vector control (pcDNA) and wound closure was monitored at 24 hours time point. D) TPC-1 cells transfected with AGR2, AGR2 (C→S) or the empty vector (pcDNA) were seeded in the upper chamber of transwells and incubated for 24 hours; the upper surface of the filter was wiped clean and cells on the lower surface were stained. Click here for file
