# Supplementary material for: Anterior gradient protein 2 promotes survival, migration and invasion of papillary thyroid carcinoma cells
Source: Mol Cancer. 2014 Jun 30;13:160. doi: 10.1186/1476-4598-13-160 (PMC4094684; doi:10.1186/1476-4598-13-160)
Supplement: Additional file 1: Figure S1 — The chemical crosslinker DSS was added to Nthy-ori 3-1 AGR2 cell suspension at final concentration of 1 mM. Monomeric and dimeric AGR2 were detected by Western blotting with AGR2 antibody. Tubulin levels were used for normalization. Figure S2. Effects of AGR2 ectopic expression on TPC-1 cell growth, migration and invasion. A) Expression levels of transfected AGR2, AGR2 (C→S) and AGR2 (E→A) in TPC-1 cells: after G418 selection, cells were lysed and blotted with the indicated antibodies. Levels of exogenous AGR2 are shown. B) TPC-1 cells transfected with AGR2, AGR2 (C→S), AGR2 (E→A) or empty vector (pcDNA) were plated and counted at different time points. Values represent the average of triplicate experiments ± standard deviations. C) A wound was introduced on confluent monolayer of TPC-1 cells transfected with AGR2, AGR2 (C→S), AGR2 (E→A) or vector control (pcDNA) and wound closure was monitored at 24 hours time point. D) TPC-1 cells transfected with AGR2, AGR2 (C→S) or the empty vector (pcDNA) were seeded in the upper chamber of transwells and incubated for 24 hours; the upper surface of the filter was wiped clean and cells on the lower surface were stained. [file 1476-4598-13-160-S1.doc]

**Anterior gradient protein 2 promotes survival, migration and invasion of papillary thyroid carcinoma cells**

Gennaro Di Maro^1^, Paolo Salerno^1^, Kristian Unger^2^, Francesca Maria Orlandella^1^, Mario Monaco^3^, Gennaro Chiappetta^3^, Gerry Thomas^2^, Malgorzata Oczko-Wojciechowska^4^, Mariorosario Masullo^5^, Barbara Jarzab^4^, Massimo Santoro^1^, Giuliana Salvatore^5^

^1^ Dipartimento di Medicina Molecolare e Biotecnologie Mediche, Università di Napoli “Federico II”, Italy.

^2^ Department of Surgery and Cancer, Hammersmith Hospital, Imperial College London, London, UK.

^3^ UOC Genomica Funzionale, Dipartimento Ricerca, Istituto Nazionale Tumori Fondazione G. Pascale- IRCCS, Napoli, Italia.

^4^ MSC Memorial Cancer Center and Institute of Oncology, Gliwice, Poland.

^5^ Dipartimento di Scienze Motorie e del Benessere, Universita’ “Parthenope”, Naples, Italy.

**Corresponding author**: Giuliana Salvatore, Dipartimento di Scienze Motorie e del Benessere, Universita’ “Parthenope”, Naples, Via Medina 40, 80133 Naples, Italy. Ph: + 39-081-7463847, Fax: +39-081-74634581. E-mail: giuliana.salvatore@uniparthenope.it.

**Supplementary Informations**


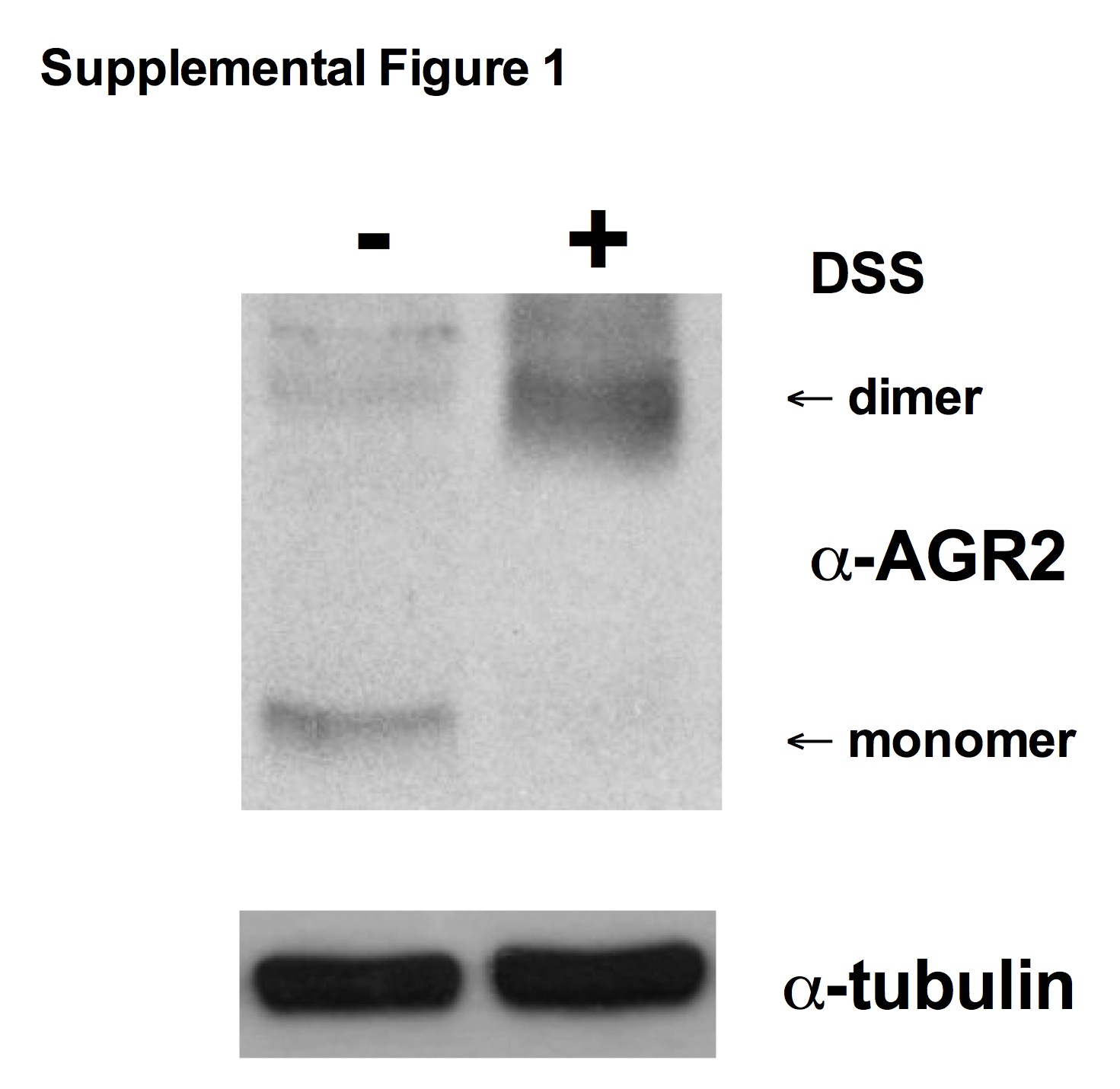


**Supplemental Figure 1**: The chemical crosslinker DSS was added to Nthy-ori 3-1 AGR2 cell suspension at final concentration of 1 mM. Monomeric and dimeric AGR2 were detected by Western blotting with AGR2 antibody. Tubulin levels were used for normalization.

**
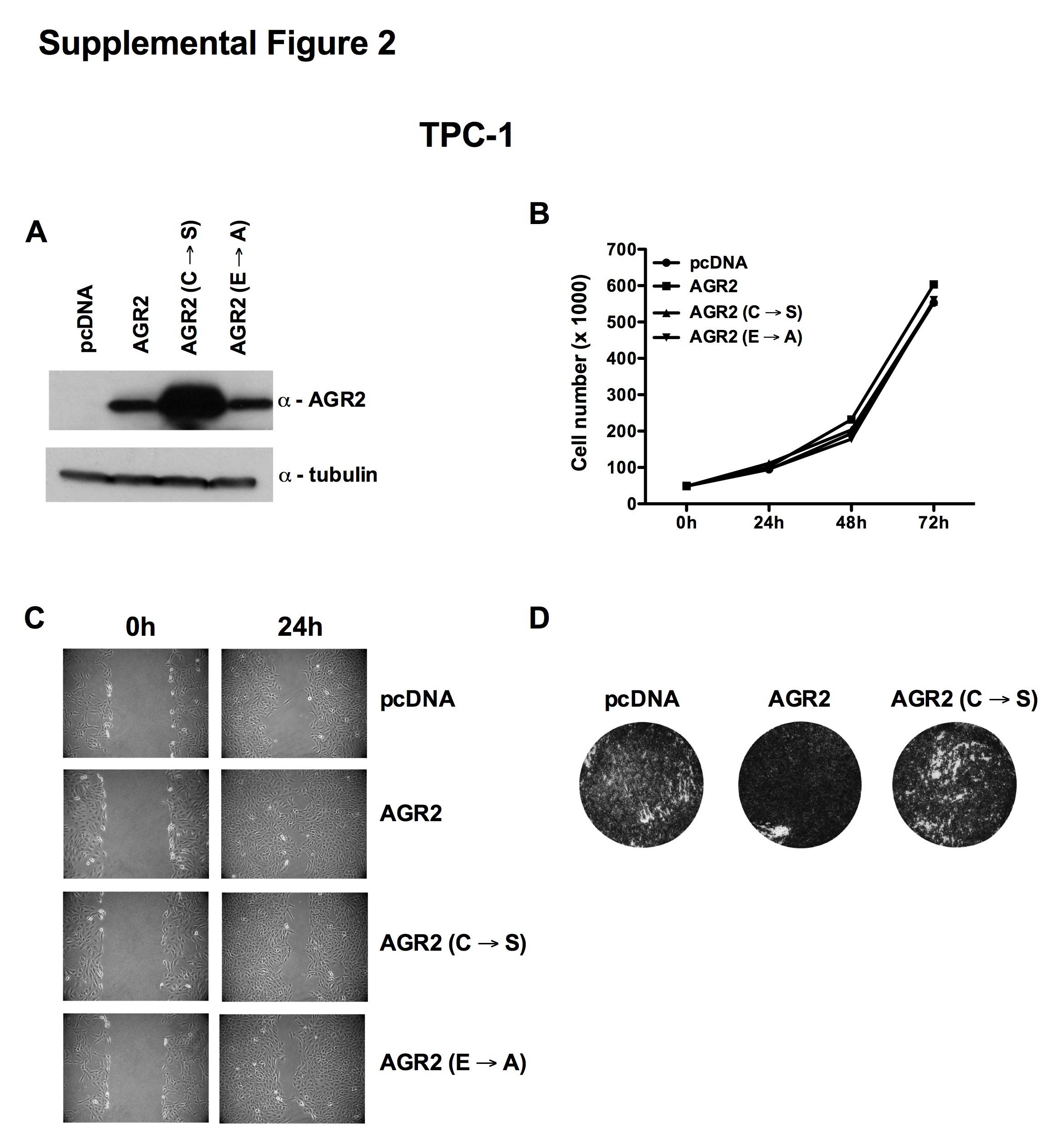
**

**Supplemental Figure 2**: **Effects of AGR2 ectopic expression on TPC-1 cell growth, migration and invasion**.

**A)** Expression levels of transfected AGR2, AGR2 (C→S) and AGR2 (E→A) in TPC-1 cells: after G418 selection, cells were lysed and blotted with the indicated antibodies. Levels of exogenous AGR2 are shown. **B**) TPC-1 cells transfected with AGR2, AGR2 (C→S), AGR2 (E→A) or empty vector (pcDNA) were plated and counted at different time points. Values represent the average of triplicate experiments ± standard deviations. **C)** A wound was introduced on confluent monolayer of TPC-1 cells transfected with AGR2, AGR2 (C→S), AGR2 (E→A) or vector control (pcDNA) and wound closure was monitored at 24 hours time point. **D**) TPC-1 cells transfected with AGR2, AGR2 (C→S) or the empty vector (pcDNA) were seeded in the upper chamber of transwells and incubated for 24 hours; the upper surface of the filter was wiped clean and cells on the lower surface were stained.
